# Proximal Phenotyping and Machine Learning Methods to Identify Septoria Tritici Blotch Disease Symptoms in Wheat

**DOI:** 10.3389/fpls.2018.00685

**Published:** 2018-05-23

**Authors:** Firuz Odilbekov, Rita Armoniené, Tina Henriksson, Aakash Chawade

**Affiliations:** ^1^Department of Plant Breeding, Swedish University of Agricultural Sciences, Alnarp, Sweden; ^2^Lantmännen Lantbruk, Svalöv, Sweden

**Keywords:** Septoria tritici blotch, wheat, proximal phenotyping, disease detection, machine learning, random forest, machine learning

## Abstract

Phenotyping with proximal sensors allow high-precision measurements of plant traits both in the controlled conditions and in the field. In this work, using machine learning, an integrated analysis was done from the data obtained from spectroradiometer, infrared thermometer, and chlorophyll fluorescence measurements to identify most predictive proxy measurements for studying Septoria tritici blotch (STB) disease of wheat. The random forest (RF) models for chlorosis and necrosis identified photosystem II quantum yield (QY) and vegetative indices (VIs) associated with the biochemical composition of leaves as the top predictive variables for identifying disease symptoms. The RF model for chlorosis was validated with a validation set (*R*^2^: 0.80) and in an independent test set (*R*^2^: 0.55). Based on the results, it can be concluded that the proxy measurements for photosystem II, chlorophyll content, carotenoid, and anthocyanin levels and leaf surface temperature can be successfully used to detect STB. Further validation of these results in the field will enable application of these predictive variables for detection of STB in the field.

## Introduction

Septoria tritici blotch (STB) caused by *Zymoseptoria tritici* is currently one of the most devastating foliar diseases of wheat in Northwestern Europe causing yield losses every year (Fones and Gurr, 2015; Chawade et al., 2018). It is a hemibiotrophic fungus which penetrates host leaves through stomata and grows very slowly in the intercellular spaces of the mesophyll cells. The latent phase varies between 14–28 days under field conditions and 9–14 days under laboratory conditions (Kema et al., 1996; Shetty et al., 2003; Keon et al., 2007). This symptomless period has been referred to as ‘biotrophic’ (Kema et al., 2000), however, after more detailed transcriptomic and metabolic analysis, this term has become debatable (Rudd et al., 2015; Sánchez-Vallet et al., 2015). After a latent period, the fungus switches to necrotrophic phase and the infected leaves become chlorotic and develop into necrotic irregularly-shaped blotches (lesions) in which fungal asexual fruiting sporulation structures called pycnidia develop (Steinberg, 2015; Kettles and Kanyuka, 2016).

A cultivar with a high level of resistance can provide an effective mode to control the disease severity, but so far, cultivars with complete resistance are not developed (Chartrain et al., 2004). STB is typically controlled by fungicides and due to the intensive chemical control, *Z. tritici* populations can rapidly evolve resistance to fungicides (Goodwin et al., 2011). For STB, best practice requires that the fungicides should be sprayed early on in the latent period, as the fungicide application has limited effectiveness in the necrotrophic phase (Fones and Gurr, 2015). Detection of STB in the latent stage can provide more efficient disease control by fungicides, thus minimizing directional selection that favors mutations encoding high level of resistance to fungicides in the *Z. tritici* populations.

Optical imaging techniques such as RGB, thermal, fluorescence, multi- and hyper-spectral imaging were applied to detect various plant diseases (Mahlein, 2016). Colonization of fungal pathogens cause multiple biochemical, physiological and morphological alterations in leaf tissue and it can be inferred from the reflectance of light at visible (VIS, 400–700 nm) and near-infrared (NIR, 700–2000 nm) regions of the electromagnetic spectrum. Hyperspectral imaging can be used to detect foliar diseases in early pathogenesis stage before visible symptoms appear (Kuska et al., 2015; Xie et al., 2016; Thomas et al., 2017). Hyperspectral imaging in VIS/NIR ranges was demonstrated as a powerful tool for detection and/or differentiation of foliar fungal diseases in barley (Kuska et al., 2015; Thomas et al., 2017), cucumber (Berdugo et al., 2014), sugar beet (Mahlein et al., 2012b), wheat (Ashourloo et al., 2014a,b; Cao et al., 2015; Iori et al., 2015), tomato (Xie et al., 2016), oilseed rape (Baranowski et al., 2015), and strawberries (Yeh et al., 2016).

Visible range is mainly influenced by leaf pigments like chlorophyll and carotenoid content and NIR is influenced by leaf structure, internal scattering processes and by leaf water content (Mahlein, 2016). Spectral vegetation indices (VIs) are mathematical equations and transformations derived from two or more wavelengths in the electromagnetic spectrum (Araus et al., 2001). Application of VIs is a common approach to investigate or identify changes in plant physiology and morphology. VIs were developed as proxies to evaluate various plant properties such as leaf area (Rouse et al., 1974), water content (Penuelas et al., 1995), and leaf pigment content (Gitelson et al., 2002; Sims and Gamon, 2002). Among the indices developed, NDVI (Normalized Difference Vegetation Index) is most commonly used as it can estimate nutrient requirements of plants and is thus used for optimizing fertilizer input in the fields (Raun et al., 2002). More than 100 VIs have been developed so far and summarized earlier (Devadas et al., 2008; Agapiou et al., 2012; Pietragalla et al., 2012; Lehnert et al., 2017).

Advances in sensor phenotyping technologies will generate big data. Therefore, extracting patterns and features from this big data requires machine learning (ML) tools (Singh et al., 2016). Application of the ML methods for prediction of various diseases from spectral reflectance data was reviewed recently (Lowe et al., 2017). Using spectral reflectance data, yellow rust in wheat was detected with quadratic discriminant analysis (Bravo et al., 2003; Lowe et al., 2017), multilayer perceptron (Moshou et al., 2004), and regression (Huang et al., 2007). While leaf rust was detected with maximum likelihood classification (Ashourloo et al., 2014b) and powdery mildew with Fishers linear discriminant analysis (Zhang et al., 2012). Thus, spectral reflectance phenotyping and ML methods hold promise for disease detection at early stages of infection.

Early detection of disease symptoms allows taking control measures to avoid further spread of pathogen and consequent yield losses. Disease monitoring methods are time-consuming, can be affected by subjective bias and expensive (Bock et al., 2010). Therefore, there is an increasing demand for innovative and reliable disease monitoring method (Bravo et al., 2003; Mahlein et al., 2012a). The aim of the present study was to evaluate the possibility of identifying disease progression stages of STB on wheat with proximal phenotyping and machine-learning.

## Materials and Methods

### Fungal Inoculation

The *Z. tritici* isolate was isolated from typical STB lesions on leaves of winter wheat collected in 2015 in a field in Lomma, Sweden. The inoculum was obtained from stock conidial suspensions of the isolate stored at -80°C in a sterile 1:1 glycerol–water solution. The fungal isolate was retrieved by adding 10 μl of the spore suspension to Petri dishes containing fresh 4-4-4 agar-malt-yeast medium (YMSA) with antibiotic Kanamycin (50 μg/ml) (Saidi et al., 2012). The isolate was spread on the medium by adding 1 ml of sterile water after 1 day of growth. Petri dishes with the isolate were incubated at 20°C with 12 h photoperiod. Conidial suspensions were prepared by first flooding the surface of the 10-day-old cultures with sterile distilled water and then by scraping the agar surface with a sterilized paint brush to release conidia. The spore concentration was measured using a Neubauer counting chamber. Thereafter, 0.1% TWEEN20 (Sigma) was added to the spore suspension and the final spore concentration was adjusted to 10^7^ spores ml^-1^.

### Plant Material

Two independent experiments were conducted under greenhouse conditions. In the first experiment (training and validation set), 10 winter wheat cultivars/breeding lines (Stigg, Oakley, Nelson, Mariboss, Kovas DS, Julius, Hereford, SW05317, SW75638, and Target) were evaluated for resistance to STB. Whereas, in the second experiment (test set) two winter wheat cultivars (Kranich and Nimbus) were evaluated for STB resistance. For both experiments, seeds were germinated for 2 days on a moist filter paper in Petri dishes. Germinated seeds were sown in plastic pots (8 cm × 8 cm × 8 cm) filled with peat substrate Blomjord Exclusive (Emmaljunga Torvmull AB, Sweden). For each genotype, two seeds were sown per pot in three replications in a randomized block design. Plants were grown in a greenhouse at 22°C (day) and 18°C (night) with a 16 h photoperiod.

### Inoculation Procedure

Seedlings were inoculated following the full emergence of the third leaf and about 21 days after planting. The conidial suspension was applied to both sides of marked second and third leaf using a flat paintbrush (bristle length 15 mm). The control plants were inoculated with water. Following inoculation, plant leaves were allowed to dry for 1 h before transferring to the humidity chamber. Plants were kept under the plastic tent at close to 100% humidity for 48 h before being returned to the greenhouse conditions.

### Disease Assessment

Disease severity was visually assessed at time-points 15, 17, 18, 20, 21, and 23 for the training and validation set and at 6, 8, 10, 13, 14, 15, and 16 days post-inoculation (dpi) for the test set. Percentage of the inoculated leaf surface (from 0 to 100%) presented the following symptoms: chlorosis [the percentage of chlorotic area (CHL)] and necrosis [the percentage of necrotic area (NEC)]. The symptoms and lesion development over the assessment period were summarized by area under the disease progress curve (AUDPC). Minitab software (Version 17.1.0) was used for statistical calculations. Differences in AUDPC were investigated with ANOVA (PROC GLM) and comparisons of means with Tukey’s test.

### Sensor Phenotyping

In the training and validation set, sensor phenotyping was done at time points 14, 15, 17, and 18 dpi for the infected plants while for the mock-inoculated plants, sensor phenotyping was done at 14 dpi. In the test set, sensor phenotyping was done for both mock-inoculated and infected plants at 6, 8, 10, 13, 14, 15, and 16 dpi. Earlier time-points were additionally included in the test set to evaluate the possibility to detect disease symptoms earlier in the disease progression with the developed computational models. A handheld active light fluorometer (FluorPen FP 100-MAX, Photon Systems Instruments, Czechia) with detachable leaf-clips was used for measuring QY (Photosystem II quantum yield). For QY measurements, for each plant, two leaf-clips were attached to the control or infected leaves and were dark adapted for 15 min prior to the measurements. Thereafter, the leaves were removed from the plants and spectral reflectivity (350–1150 nm) of the leaves were recorded with a resolution of 1 nm with a handheld spectroradiometer sensor Apogee PS-100 (Apogee Instruments, Inc., United States) using a reflectance probe with an internal light source (AS-003, Apogee Instruments, Inc., United States). The spectroradiometer was calibrated against a white reference standard Apogee AS-004 (Apogee Instruments, Inc., United States) prior to the measurements. The leaf temperature was measured with a infrared thermometer Apogee MI-210 (Apogee Instruments, Inc., United States). Finally, the leaves were scanned with Epson Perfection V200 scanner.

### Spectral Data Analysis and Machine Learning

The obtained raw spectral reflectance data files were analyzed further to remove noise, detect outliers and calculate VIs using the open-source software Specalyzer^1^ and the hsdar R package (Lehnert et al., 2017). Data quality of the spectral files was inspected manually in Specalyzer. Each replicate consisting of two plants was considered as a sample. For spectral measurements from all samples, areas around the edges of the spectra were trimmed due to low signal-to-noise ratio and the region from 420 to 1000 nm was retained for further analysis. Finally, 119 previously known VIs were estimated from the spectral data in Specalyzer for further analysis (Supplementary File 1). Thus, in total, 121 variables were obtained for each sample consisting of 119 VIs, QY, and leaf surface temperature. PCA (principal component analysis) was performed in the software Simca 14.1 (Umetrics, Sweden) and the data was scaled by unit variance (UV) scaling method for PCA.

Random forest (RF) regression models were built from the training set with 121 samples from eight cultivars/breeding lines and was validated on the validation set of 30 samples from two lines (SW75638 and Target) consisting 6 uninfected and 24 infected samples. Recursive feature elimination algorithm (RFE) from the R package Caret (Kuhn et al., 2016) was used for feature selection with parameters: function “rfFuncs,” method “repeatedcv” and repeats 10. Separate prediction models were thereafter built with the features selected with RFE for chlorosis and necrosis using the R package Caret. Common parameters for building the models were the variables selected by RFE, eight cultivars from the training set, ntrees 2000, resampling method “repeatedcv,” number 10, repeats 10 and importance “True.” Percentage of chlorosis and necrosis were used as scoring parameters for model training and testing. The models were tested on a test set consisting of 94 samples with equal number of infected and uninfected samples from the winter wheat cultivars Kranich and Nimbus.

## Results

### Genotype Variation for STB Severity

**FIGURE 1:**
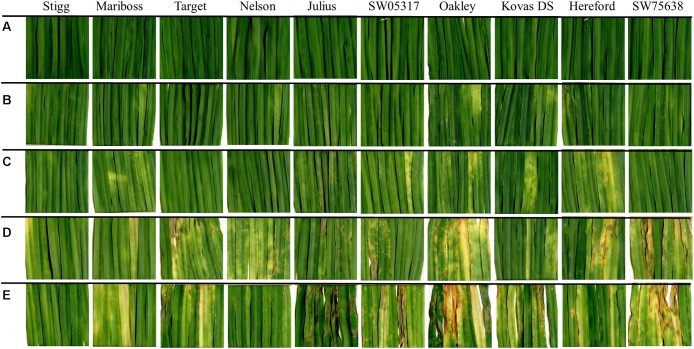
Septoria tritici blotch (STB) symptoms on 10 winter wheat cultivars at **(A)** 14 dpi control; **(B)** 14 dpi infected; **(C)** 15 dpi infected; **(D)** 16 dpi infected; and **(E)** 18 dpi infected. Cultivars are sorted based on the necrotic symptoms.

**FIGURE 2:**
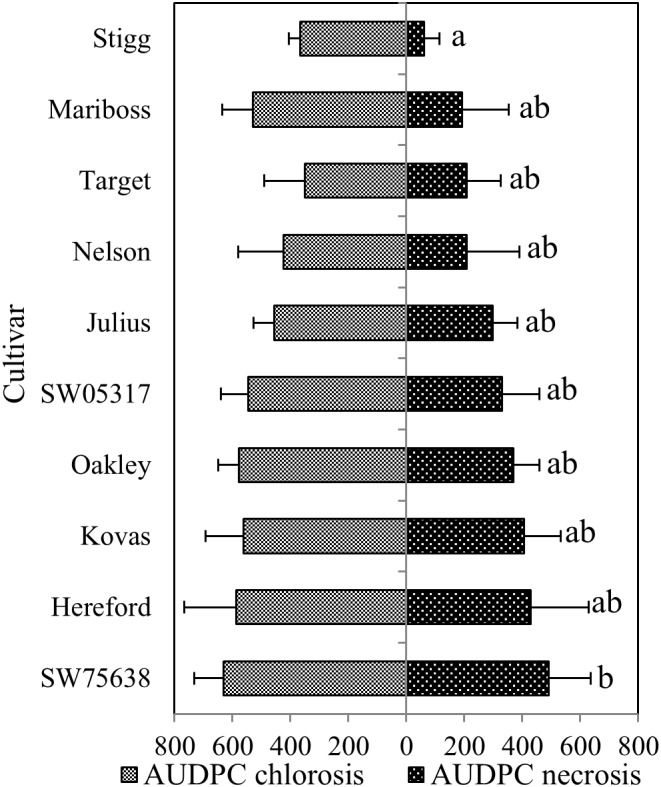
The area under the disease progress curve (AUDPC) for STB among the 10 different cultivars and breeding lines. Means that do not share a letter are significantly different at *p* < 0.05 (Tukey multiple comparison test). Ordered based on the necrotic symptoms.

**FIGURE 3:**
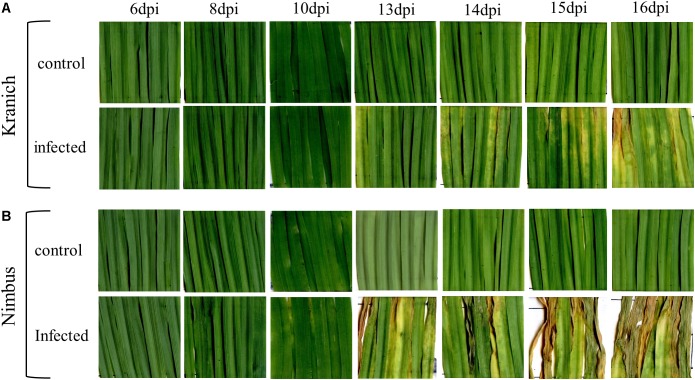
Symptoms of STB on two winter wheat cultivars. **(A)** Kranich, **(B)** Nimbus, with representative pictures of the disease symptoms after 6, 8, 10, 13, 14, 15, and 16 dpi.

**FIGURE 4:**
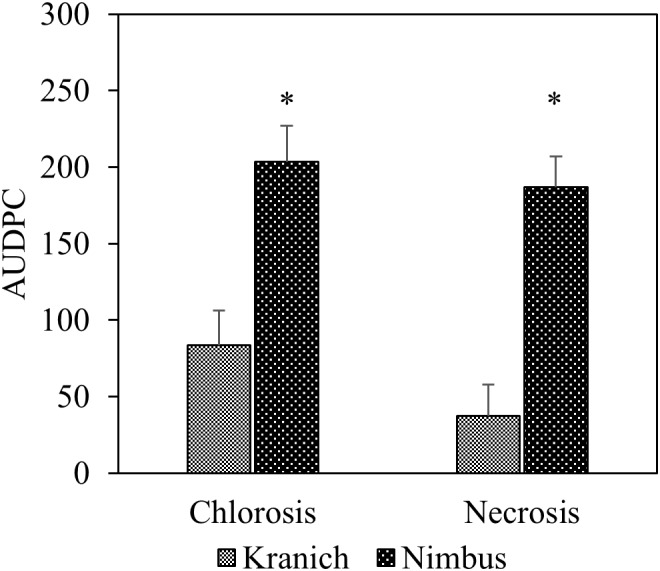
The area under the disease progress curve (AUDPC) for STB for the two cultivars Kranich and Nimbus (two sample *t*-test, ^∗^*p* < 0.01).

In the training set, 10 genotypes showed good variation in STB severity across all time points upon infection (**Figure 1**). Cultivar Stigg had less chlorotic and necrotic symptoms compared to the cultivar Hereford and the breeding line SW75638. The chlorotic symptoms were visible by 14 dpi but were more pronounced by 16 dpi in most genotypes. Necrotic symptoms appeared by 16 dpi in the susceptible genotype but Stigg did not show many necrotic symptoms even at 18 dpi. AUDPC was calculated based on scoring for chlorosis and necrosis at all timepoints to quantify resistance (**Figure 2**). A significant difference (*p* < 0.01) among the 10 winter wheat genotypes was observed in AUDPC of NEC. Target, Stigg, and Nelson revealed highest level of resistance and the most susceptible cultivars in this experiment were Hereford and SW75638 (**Figure 2**). A significant correlation (*r* = 0.86, *p* < 0.001) was also found between the AUDPC of CHL and NEC. In the test set, clear differences in the chlorotic and necrotic symptoms were observed among the two winter wheat cultivars Nimbus and Kranich (**Figure 3**). Disease symptoms appeared much earlier in Nimbus (at 13 dpi) compared to Kranich. Quantification of CHL and NEC by AUDPC in the test set showed significant differences between the two cultivars (*p* < 0.01, **Figure 4**). Cultivar Kranich exhibited a higher level of resistance compared to Nimbus in both STB disease development stages (CHL and NEC).

### Multivariate Analysis

**FIGURE 5:**
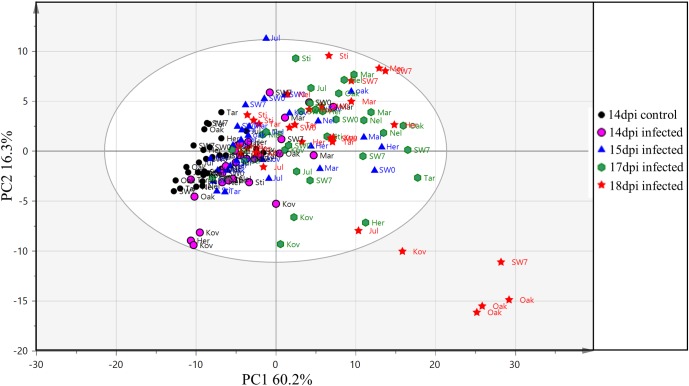
Principal component analysis (PCA) from the vegetation indices, QY and leaf temperature of 10 winter wheat cultivars and breeding lines (training and validation set) after 14, 15, 17, and 18 days post-infection. Elliptical region represents the normal operating area by Hotelling’s T2 tolerance with 95% confidence. Sti: Stigg; Oak: Oakley; Nel: Nelson; Mar: Mariboss; Kov: Kovas DS; Jul: Julius; Her: Hereford; SW0: SW05317; SW7: SW75638; and Tar: Target.

**FIGURE 6:**
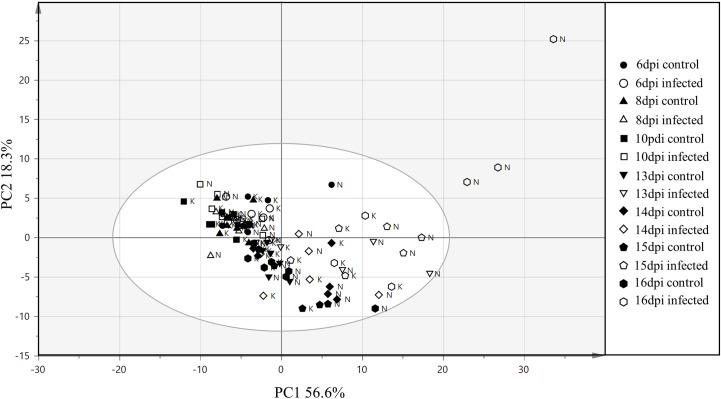
Principal component analysis from the vegetation indices, QY and leaf temperature of two winter wheat cultivars (test set) N: Nimbus and K: Kranich across different time points. Elliptical region represents the normal operating area by Hotelling’s T2 tolerance with 95% confidence.

Clustering of samples from the training and the test set were studied by PCA of 121 variables consisting of 119 VIs, leaf surface temperature and QY measurements from each sample. In the PCA of the training set, the first and the second components explained 60.2 and 16.3% of the variation respectively (**Figure 5**). Most of the samples from the early time points clustered together (14 and 15 dpi), whereas, samples from the later time points (17 and 18 dpi) were more scattered. The first component explained the variability in the disease progression over time while the second component explained the inter-cultivar variation during disease progression. Thus, disease progression over time was the major variability in the data explained by the PCA. In **Figure 5**, it can be observed that the variability in the data increases with the disease progression. The control samples at 14 dpi have the lowest variability and are thus relatively tightly clustered followed by increasing separation among the infected samples from 14 to 18 dpi. The results from PCA indicates that the 10 cultivars in the training set have physiological differences in their response to the infection which is also corroborated by disease symptoms evaluated with AUDPC analysis (**Figure 2**). In the PCA plot from the test set, the two PCA components explained 56.6 and 18.3% variation respectively (**Figure 6**). Similar to the PCA from the training set, in the test set, the first component explained the variability in the disease progression over time, additionally, some separation was also observed among the control samples as the control samples from the later time-points separated from earlier time-points. This suggests physiological differences in the control plants occurring over a duration of 10 days (6–16 dpi). Among the infected plants, sample separation in Nimbus (susceptible) is detected at 13 dpi whereas in Kranich (resistant) the separation was at 15 dpi. Also, a clear and distinct separation of Nimbus samples at 16 dpi is observed. This indicates distinct differences in the physiological status of Kranich and Nimbus genotypes upon STB infection and these differences become apparent after 13 dpi with the sensor measurements.

**FIGURE 7:**
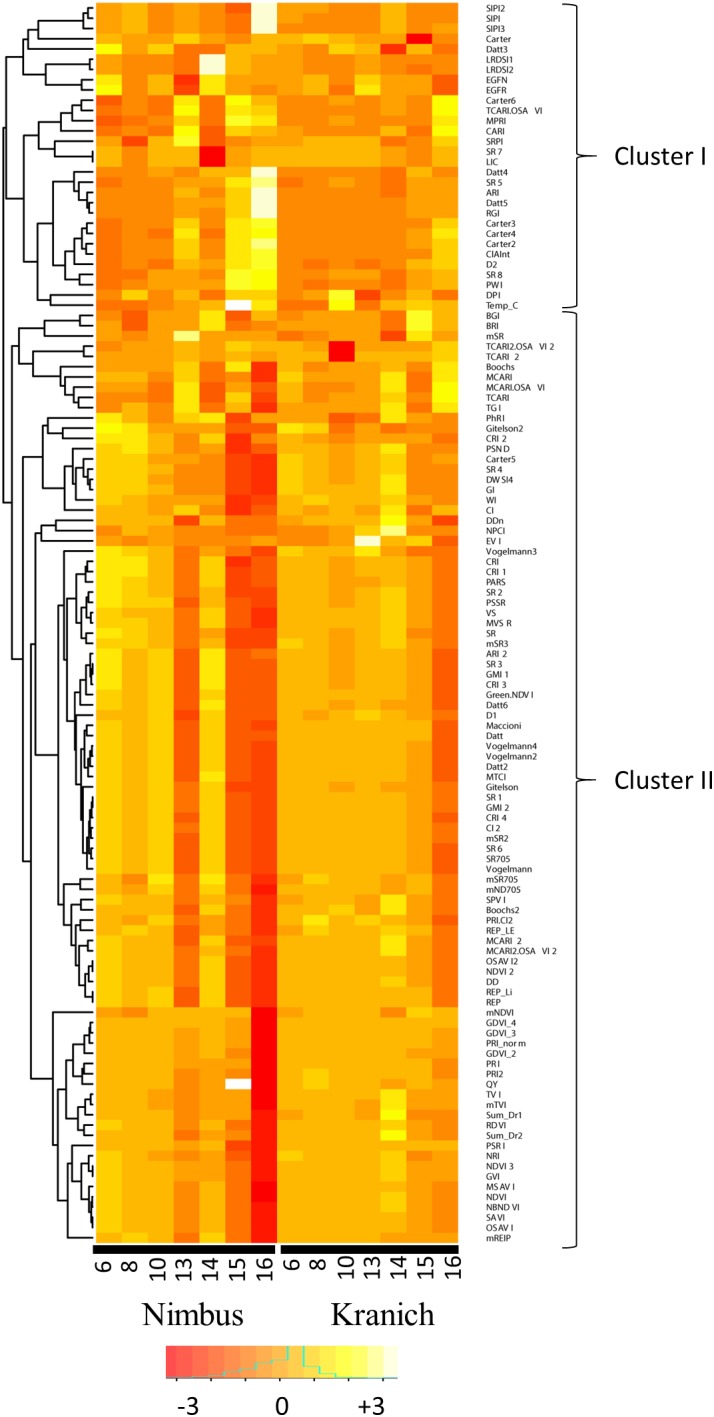
The relative intensities of various vegetation indices of two winter wheat cultivars after infection with STB across different time points.

A heatmap was prepared for the test set from the relative intensities of the sensor phenotyping data obtained from the ratio of the data from the infected plants to that of the mock-inoculated plants (**Figure 7**). Based on the dendrogram, two clusters were obtained at the highest level of tree branching, cluster-I consisted of 30 variables and cluster II 91 variables. As can be seen from the heatmap, lower relative intensities were recorded for several VIs in cluster-II at the later time-points in both cultivars and distinctly lower intensities were observed in the susceptible cultivar Nimbus. Variables in cluster-II negatively correlate with STB symptoms as the intensities of these variables decrease with increase in necrosis. Several variables in cluster-II were affected by necrosis already upon the first visible symptoms of necrosis at 13 dpi in the susceptible cultivar Nimbus. In the resistant cultivar Kranich, relatively less pronounced changes in intensities of variables from cluster-II were seen. Furthermore, unlike in Nimbus, variables in cluster-II were not affected at earlier time-points in Kranich which is in accordance with the delayed necrosis symptoms observed in Kranich.

**FIGURE 8:**
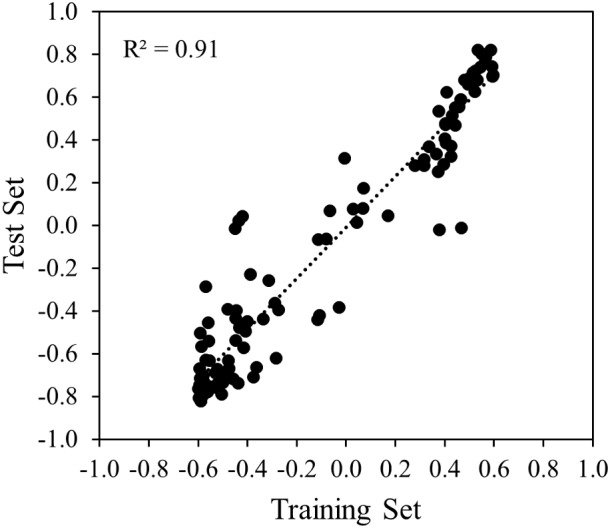
Scatter plot and the simple linear regression line between training and test set.

Correlation analysis of the sensor data was done between the training and the test set to analyze the reproducibility of the measurements over time and genotypes. At first, for each experiment, correlation analysis was done separately for each of the 121 variables and the respective CHL measurement of the sample, thereafter, correlations obtained for each variable from the two experiments were compared. A high coefficient of determination (*R*^2^ = 0.91) was obtained indicating good reproducibility of the measurements under similar conditions across time and genotypes (**Figure 8**).

### STB Detection With Random Forest

**FIGURE 9:**
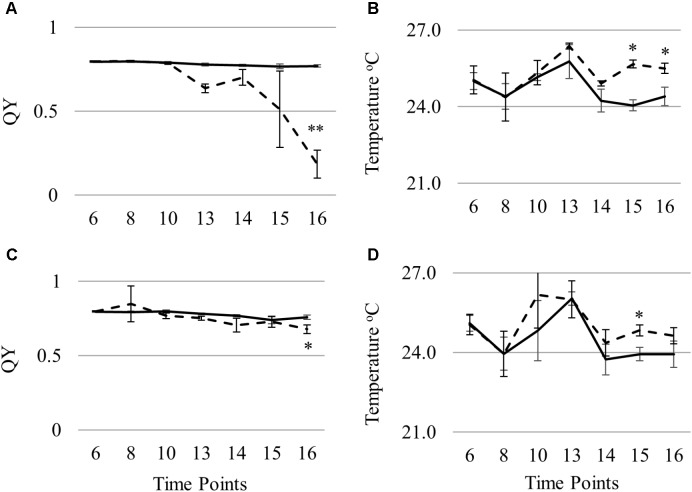
Differences in QY **(A,C)** and leaf surface temperature **(B,D)** in Nimbus **(A,B)** and Kranich **(C,D)** across different time points (two sample *t*-test, ^∗^*p* < 0.05).

To identify and evaluate key predictive variables for STB infection, automated feature selection was done followed by building RF models with the selected features. For chlorosis, the feature elimination algorithm RFE identified four variables QY, D2 (derivative index), LRDSI1 (leaf rust disease severity index) and LRDSI2 as most predictive. While for necrosis, five variables namely QY, MCARI2/OSAVI2, ARI (anthocyanin reflectance index), SR8 (simple ratio 8) and D1 were identified by the RFE algorithm as important. RF regression models were developed separately for CHL and NEC from the training set and the selected variables. The percentage of variation explained by the models was 45.41% for chlorosis and 21.04% for necrosis with a mean of squared residuals of 581 and 284 respectively. The variables QY was identified as predictive in both chlorosis and necrosis models and had significantly different (*p* < 0.05) levels in the infected plants compared to the mock-inoculated plants (**Figure 9**). Leaf surface temperature was not selected as a predictive variable although there were significant differences in the surface temperature of the infected and mock-inoculated plants (**Figure 9**).

**FIGURE 10:**
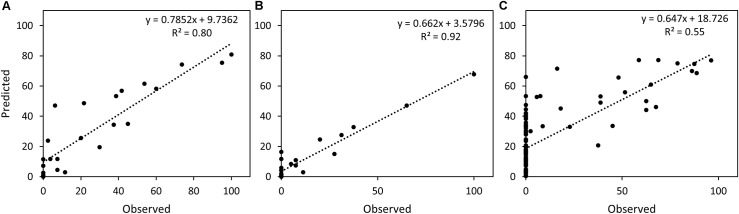
Scatter plot and the simple linear regression line between predicted and observed STB infection scores. **(A)** Chlorosis in the validation set, **(B)** necrosis in the validation set, **(C)** chlorosis in the test set.

The two RF models were tested on a validation set consisting of 30 samples from the genotypes SW75638 and Target and an independent test set of 94 samples from two cultivars Kranich and Nimbus. The samples were predicted separately with the RF models built for CHL and NEC. A simple linear regression was calculated to estimate the relationship between the observed and the predicted STB infection. For the validation set, chlorosis was predicted with *R*^2^: 0.80 and necrosis with *R*^2^: 0.92, while for the test set, chlorosis was predicted with *R*^2^: 0.55 (**Figure 10**).

## Discussion

In the present work, sensor phenotyping with spectral reflectance, chlorophyll fluorescence, and leaf temperature was performed to evaluate the possibility of detecting different STB developmental stages. In this study, the number of indices affected by the disease increased upon disease progression (**Figure 7**), an observation which was also previously discussed (Ashourloo et al., 2014b). This is due to the magnitude of changes in the leaf morphology and physiology brought upon by disease progression. Sensor phenotyping clearly separated control and infected plants based on the progression of the disease (**Figures 5**, **6**). This separation is influenced by the underlying genetic resistance of the genotypes to STB.

In this work, different indices were identified as top predictive indices for chlorosis and necrosis except for QY which was common in both. This can be due to the distinctly different leaf composition in these two stages. QY (PSII) was affected by STB in the susceptible genotype Nimbus at an early stage of disease progression but was not affected to the same extent in Kranich (**Figure 9**). Previously, chlorophyll fluorescence kinetics were studied for powdery mildew and leaf rust infection in wheat and early detection of infection was possible with chlorophyll fluorescence measurements but not with NDVI (Kuckenberg et al., 2008). In this work, leaf infrared temperature was not selected as a top predictive variable by RF. Leaf temperature was significantly different in the control and infected plants of the two cultivars from the test set at 15 dpi. In the susceptible cultivar Nimbus, statistically significant difference of 1.5°C (*p* < 0.05) was observed between the control and the infected plants at time point 15 dpi with higher temperature recorded from the infected plants (**Figure 9**). These results confirm the results from a previous work where the canopy temperature measured with an infrared thermometer positively correlated (*r* = 0.48–0.74) with STB coverage in the field (Eyal and Blum, 1989).

The top predictive VIs for chlorosis were D2, LRDSI1, and LRDSI2. The derivative index D1 and D2 correlated well with the natural steady state chlorophyll fluorescence emission by photosystem I and II in the range 639–730 nm (Zarco-Tejada et al., 2003). Both LRDSI1 and LRDSI2 were developed for detecting wheat leaf rust with prediction accuracies of >85% (Ashourloo et al., 2014a). Thus, the predictive VIs for chlorosis detect the levels of chlorophyll fluorescence and anthocyanin levels in the leaves.

Top predictive VIs for necrosis were MCARI2.OSAVI2, ARI, SR8, and D1. In the previous work, MCARI2.OSAVI2 was developed for measuring chlorophyll content while tolerating leaf area index (Wu et al., 2008). ARI was developed for estimating anthocyanin reflectance in senescing and stressed leaves (Gitelson et al., 2007). SR8 was developed to estimate carotenoid content in conifer forest (Hernández-Clemente et al., 2012). Thus the top predictive indices for necrosis identified in this work suggests differing levels of chlorophyll, anthocyanin, and carotenoid content in the infected leaves.

Spectral reflectance was previously used to develop spectral indices for detection of different plant diseases. In wheat, leaf rust was detected for plants in a controlled environment with the VIs NBNDVI, NDVI, PRI, GI, and RVSI with an accuracy of over 60% (Ashourloo et al., 2014b). Ashourloo et al. (2014a) developed two new VIs LRDSI1 and LRDSI2 to detect leaf rust in a controlled environment with the *R*^2^: 0.9. By proximal and airborne hyperspectral phenotyping, Huang et al. (2007) identified photochemical reflectance index (PRI) as the most predictive VI (*R*^2^: 0.97) for yellow rust detection in wheat. Cao et al. (2015) studied 17 VIs for prediction of powdery mildew in wheat under field conditions and reported that difference vegetation index (DVI), triangular vegetation index (TVI) and the area of red edge peak significantly correlated with powdery mildew severity. The RF models developed in this work for detection of STB utilize several variables and thus provide a possibility to uniquely detect STB in wheat with various sensors. STB was detected by NDVI and land surface temperature using satellite imaging with MODIS (moderate-resolution imaging spectroradiometer) and the spatial modeling conducted with linear regression trees and boosted regression trees was suggested as a promising approach for studying STB spread using satellite imaging (Wakie et al., 2016). In this work, NDVI was not detected as a top predictive index, however, the predictive VIs identified in this work can be further validated in the field conditions by proximal and remote sensing.

Further work with parallel measurements of different diseases with various sensors under both greenhouse and field conditions is required to understand the overlap of various predictive VIs.

Septoria tritici blotch resistance traits can be further combined with STB escape and tolerance traits to further reduce the disease progression and maintain yields under disease pressure. Some of the traits identified for STB escape are early vigor, growth rate, plant height, leaf length, leaf spacing, prostrate leaves, leaf insertion angle, flag leaf emergence, and heading time (Arraiano et al., 2009; Brown et al., 2015). Disease tolerance can be explained by the maintenance of yield in spite of the disease pressure. The tolerance trait was studied in a susceptible wheat cultivar ‘Miriam’ which maintained yield even under disease pressure by increasing photosynthesis in the residual green area of the infected leaves (Kuckenberg et al., 2008). Using affordable high-throughput phenotyping (Armoniené et al., 2018), and genotyping, the escape and tolerance traits can be combined with the resistance traits for developing improved wheat varieties. Furthermore, the sensor data can be integrated with transcriptomics, proteomics, and metabolomics data to improve our understanding of the underlying biological mechanisms. RF was selected in this work as it is equally effective for classification and regression (Díaz-Uriarte and Alvarez de Andrés, 2006) and has been successfully used earlier for disease detection in plants (Chawade et al., 2016). RF is efficient at identifying predictive information from data integrated from various sources and thus it can be explored further for systems-level understanding of responses of plants to various stresses.

## Conclusion

This work has resulted in identifying top predictive indices for detecting STB. Different predictive variables were selected by the RF model for prediction of chlorosis and necrosis in leaves. From this work, it can be concluded that precision phenotyping with proximal sensors holds the potential for detecting STB and further validation of the identified indices in the field conditions will enable implementation of these precision phenotyping techniques in the field for detection of STB.

## Author Contributions

AC planned and designed the project. TH recommended the genotypes. FO and RA performed the experiments. AC and FO analyzed the data. All authors interpreted the results and contributed to the writing.

## Conflict of Interest Statement

The authors declare that the research was conducted in the absence of any commercial or financial relationships that could be construed as a potential conflict of interest.

## Footnotes

1 www.specalyzer.org
