# Supplementary material for: Proximal Phenotyping and Machine Learning Methods to Identify Septoria Tritici Blotch Disease Symptoms in Wheat
Source: Front Plant Sci. 2018 May 23;9:685. doi: 10.3389/fpls.2018.00685 (PMC5974968; doi:10.3389/fpls.2018.00685)
Supplement: Supplementary file 1 [file Table_1.pdf]

**Supplementary File 1**: List of vegetation indices used in this work.

Vegetation indices used in the study (adapted from (Main et al., 2011; Agapiou et al., 2012; Lehnert et al., 2016))

| No. | Abbreviation | Name | Formula | Reference |
|---|---|---|---|---|
| 1 | ARI | Anthocyanin Reflectance Index | $(1/R_{550}) - (1/R_{700})$ | (Gitelson et al., 2001) |
| 2 | ARI2 | Anthocyanin Reflectance index 2 | $R_{800}(1/R_{550}) - (1/R_{700})$ | (Gitelson et al., 2001) |
| 3 | BGI | Blue Green Pigment Index | $R_{450}/R_{550}$ | (Zarco-Tejada et al., 2005) |
| 4 | SB703 | Single Band 703 Boochs | $D_{703}$ | (Boochs et al., 1990) |
| 5 | SB720 | Single Band 720 Boochs 2 | $D_{720}$ | (Boochs et al., 1990) |
| 6 | BRI | Browning Reflectance Index | $R_{450}/R_{690}$ | (Zarco-Tejada et al., 2005) |
| 7 | CARI | Chlorophyll Absorption Ratio Index | $R_{700} * \text{abs}(a * {}_{670} + R_{670} + b)/R_{670} * (\alpha^2 + 1)^{0.5}$<br>$\alpha = (R_{700} - R_{550})/150$<br>$b = R_{550} - (550 * \alpha)$ | (Kim et al., 1994) |
| 8 | Ctr | Carter | $R_{695}/R_{420}$ | (Carter, 1994) |
| 9 | Ctr2 | Carter 2 | $R_{695}/R_{760}$ | (Carter, 1994) |
| 10 | Ctr3 | Carter 3 | $R_{605}/R_{760}$ | (Carter, 1994) |
| 11 | Ctr4 | Carter 4 | $R_{710}/R_{760}$ | (Carter, 1994) |
| 12 | Ctr5 | Carter 5 | $R_{695}/R_{670}$ | (Carter, 1994) |
| 13 | Ctr6 | Carter 6 | $R_{550}$ | (Carter, 1994) |
| 14 | CI | Coloration Index | $R_{675} * R_{690}/R^2_{683}$ | (Zarco-Tejada et al., 2003) |
| 15 | CI2 | Coloration Index 2 | $R_{760}/R_{700} - 1$ | (Gitelson et al., 2003) |
| 16 | ClAInt | - | $\int_{600nm}^{735nm} R$ | (Oppelt and Mauser, 2004) |

| | | | | |
|---|---|---|---|---|
| 17 | CRI | Carotenoid Reflectance Index | $(1/R_{510}) - (1/R_{550})$ | (Gitelson et al., 2002) |
| 18 | CRI1 | Carotenoid Reflectance Index 1 | $1/R_{515} - 1/R_{550}$ | (Gitelson et al., 2003) |
| 19 | CRI2 | Carotenoid Reflectance Index 2 | $1/R_{515} - 1/R_{770}$ | (Gitelson et al., 2003) |
| 20 | CRI3 | Carotenoid Reflectance Index 3 | $1/R_{515} - 1/R_{550} * R_{770}$ | (Gitelson et al., 2003) |
| 21 | CRI4 | Carotenoid Reflectance Index 4 | $1/R_{515} - 1/R_{700} * R_{770}$ | (Gitelson et al., 2003) |
| 22 | D1 | Derivative index | $D_{730}/D_{706}$ | (Zarco-Tejada et al., 2003) |
| 23 | D2 | Derivative index | $D_{705}/D_{722}$ | (Zarco-Tejada et al., 2003) |
| 24 | Datt | Datt | $(R_{850} - R_{710})/(R_{850} - R_{680})$ | (Datt, 1999) |
| 25 | Datt2 | Datt 2 | $R_{850}/R_{710}$ | (Datt, 1999) |
| 26 | Datt3 | Datt 3 | $D_{754}/D_{704}$ | (Datt, 1999) |
| 27 | Datt4 | Datt 4 | $R_{672}/(R_{550} * R_{708})$ | (Datt, 1998) |
| 28 | Datt5 | Datt 5 | $R_{672}/R_{550}$ | (Datt, 1998) |
| 29 | Datt6 | Datt 6 | $R_{860}/(R_{550} * R_{708})$ | (Datt, 1998) |
| 30 | DD | Double Difference Index | $(R_{749} - R_{720}) - (R_{701} - R_{672})$ | (le Maire et al., 2004) |
| 31 | DDn | new Double Difference Index | $2 * (R_{710} - R_{660} - R_{760})$ | (le Maire et al., 2008) |
| 32 | DPI | Double Peak Index | $(D_{688} - D_{710})/D^2_{697}$ | (Zarco-Tejada et al., 2003) |
| 33 | DWSI4 | Disease water stress index 4 | $R_{550}/R_{680}$ | (Apan et al., 2004) |
| 34 | EGFN | Edge green first derivative normalized difference | $(\max(D_{650:750}) - \max(D_{500:550}))/ (\max(D_{650:750}) + \max(D_{500:550}))$ | (Peñuelas et al., 1994) |
| 35 | EGFR | Edge green first derivative ratio | $\max(D_{650:750})/ \max(D_{500:550})$ | (Peñuelas et al., 1994) |
| 36 | EVI | Enhanced Vegetation Index | $2.5 * ((R_{800} - R_{670})/(R_{800} - (6 * R_{670}) - (7.5 * R_{475}) + 1))$ | (Huete et al., 1997) |
| 37 | GDVI2 | Green Difference Vegetation Index 2 | $(R^2_{800} - R^2_{680})/(R^2_{800} + R^2_{680})$ | (Wu, 2014) |
| 38 | GDVI3 | Green Difference Vegetation Index 3 | $(R^3_{800} - R^3_{680})/(R^3_{800} + R^3_{680})$ | (Wu, 2014) |

| | | | | |
|---|---|---|---|---|
| 39 | GDVI4 | Green Difference Vegetation Index 4 | $(R^4_{800} - R^4_{680})/(R^4_{800} + R^4_{680})$ | (Wu, 2014) |
| 40 | GI | Greenness Index | $R_{554}/R_{677}$ | (Smith et al., 1995) |
| 41 | - | Gitelson | $1/R_{700}$ | (Gitelson et al., 1999) |
| 42 | - | Gitelson 2 | $(R_{750} - R_{800})/(R_{695} - R_{740}) - 1$ | (Gitelson et al., 2003) |
| 43 | GMI1 | Gitelson and Merzlyak Index 1 | $R_{750}/R_{550}$ | (Gitelson and Merzlyak, 1998) |
| 44 | GMI2 | Gitelson and Merzlyak Index 2 | $R_{750}/R_{700}$ | (Gitelson and Merzlyak, 1998) |
| 45 | Green NDVI | Green Normalized Difference Vegetation Index | $(R_{800} - R_{550})/(R_{800} + R_{550})$ | (Gitelson et al., 1996) |
| 46 | GVI | Greenness Vegetation Index | $(R_{682} - R_{553})/(R_{682} + R_{553})$ | (Gandia et al., 2004) |
| 47 | LIC | Lichtenthaler indices | $R_{440}/R_{690}$ | (Lichtenthaler et al., 1996) |
| 48 | LRDSI1 | Leaf Rust Disease Severity Index 1 | $6.9*(R_{605}/R_{455})-1.2$ | (Ashourloo et al., 2014) |
| 49 | LRDSI2 | Leaf Rust Disease Severity Index 2 | $4.2 * (R_{695}/R_{455}) - 0.38$ | (Ashourloo et al., 2014) |
| 50 | - | Maccioni | $(R_{780} - R_{710})/(R_{780} - R_{680})$ | (Maccioni et al., 2001) |
| 51 | MCARI | Modified Chlorophyll Absorption in Reflectance Index | $((R_{700} - R_{670}) - 0.2 * (R_{700} - R_{550})) * (R_{700}/R_{670})$ | (Daughtry et al., 2000) |
| 52 | MCARI2 | Modified Chlorophyll Absorption in Reflectance Index 2 | $((R_{750} - R_{705}) - 0.2 * (R_{750} -R_{550})) * (R_{750}/R_{705})$ | (Wu et al., 2008) |
| 53 | MCARI2/OSAVI2 | MCARI2/OSAVI2 | MCARI2/OSAVI2 | (Wu et al., 2008) |
| 54 | MCARI/OSAVI | MCARI2/OSAVI | MCARI2/OSAVI | (Daughtry et al., 2000) |
| 55 | mNDVI | Modified NDVI | $(R_{800} - R_{680})/(R_{800} + R_{680} – 2 * R_{445})$ | (Sims and Gamon, 2002) |
| 56 | mNDVI2 | Modified NDVI 2 | $(R_{750} - R_{705})/( R_{750} + R_{705} -2 * R_{445})$ | (Sims and Gamon, 2002) |
| 57 | MPRI | Modified Photochemical Reflectance Index | $(R_{515} - R_{530})/(R_{515} + R_{530})$ | (Hernández-Clemente et al., 2011) |
| 58 | mREIP | Modified Red-Edge Inflection Point | Modified REIP with inverted Gaussian fit on reflectance | (Miller et al., 1990) |
| 59 | mSAVI | Modified Soil Adjusted Vegetation Index | $0.5 * (2 * R_{800} + 1- ((2 * R_{800} + 1)^2 – 8 * (R_{800} - R_{670}))^{0.5})$ | (Qi et al., 1994) |
| 60 | mSR | Modified Simple Ratio | $(R_{800}–R_{445})/(R_{680}–R_{445})$ | (Sims and Gamon, 2002) |

| | | | | |
|---|---|---|---|---|
| 61 | mSR2 | Modified Simple Ratio 2 | $(R_{750}/R_{705})$ -1/$(R_{750}/R_{705}) + 1)^{0.5}$ | (Chen and Cihlar, 1996) |
| 62 | mSR3 | Modified Simple Ratio 3 | $(R_{800}/(R_{670} - 1))/ (R_{800}/(R_{670} + 1))^{0.5}$ | (Chen and Cihlar, 1996) |
| 63 | mSR705 | Modified Simple Ratio 705 | $(R_{750} - R_{445})/(R_{705}-R_{445})$ | (Sims and Gamon, 2002) |
| 64 | MTCI | MERIS Terrestrial Chlorophyll Index | $(R_{754} - R_{709})/(R_{709} - R_{681})$ | (Dash and Curran, 2004) |
| 65 | mTVI | Modified Triangular Vegetation Index | $1.2 * (1.2 * (R_{800} - R_{550}) - 2.5 * (R_{670} - R_{550}))$ | (Haboudane et al., 2004) |
| 66 | MVSR | Modified Vegetation Stress Ratio | $R_{723}/R_{700}$ | (White et al., 2008) |
| 67 | NBNDVI | Narrow-Band Normalised Difference Vegetation Index | $(R_{850} - R_{680})/(R_{850} + R_{680})$ | (Thenkabail et al., 2000) |
| 68 | NDVI | Normalized Difference Vegetation Index | $(R_{800} - R_{680})/(R_{800} + R_{680})$ | (Tucker, 1979) |
| 69 | NDVI2 | Normalized Difference Vegetation Index 2 | $(R_{750} - R_{705})/(R_{750} + R_{705})$ | (Gitelson and Merzlyak, 1994) |
| 70 | NDVI3 | Normalized Difference Vegetation Index 3 | $(R_{682} - R_{553})/(R_{682} + R_{553})$ | (Gandia et al., 2004) |
| 71 | NPCI | Normalized Pigment Chlorophyll Index | $(R_{680} - R_{430})/(R_{680} + R_{430})$ | (Peñuelas et al., 1994) |
| 72 | NRI | Nitrogen Reflectance Index | $(R_{570} - R_{670})/(R_{570} + R_{670})$ | (Filella et al., 1995) |
| 73 | OSAVI | Optimized Soil Adjusted Vegetation Index | $(1 + 0.16) * (R_{800}-R_{670})/(R_{800} + R_{670} + 0.16)$ | (Rondeaux et al., 1996) |
| 74 | OSAVI2 | Optimized Soil Adjusted Vegetation Index 2 | $(1 + 0.16) * (R_{750}-R_{705})/(R_{750} + R_{705} + 0.16)$ | (Wu et al., 2008) |
| 75 | RARS | Ratio Analysis of Reflectance Spectra | $R_{746}/R_{513}$ | (Chappelle et al., 1992) |
| 76 | PhRI | Physiological Reflectance Index | $(R_{550} - R_{531})/(R_{550} + R_{531})$ | (Gitelson et al., 2001) |
| 77 | PRI | Photochemical Reflectance Index | $(R_{531} - R_{570})/(R_{531} + R_{570})$ | (Gamon et al., 1997) |
| 78 | PRI2 | Photochemical Reflectance Index 2 | $(R_{570} - R_{539})/(R_{570} + R_{539})$ | (Filella et al., 1996) |
| 79 | PRI*CI2 | PRI*CI2 | PRI*CI2 | (Garrity et al., 2011) |
| 80 | PRI_norm | Normalized PRI | $PRI * (-1)/(RDVI * R_{700}/R_{670})$ | (Zarco-Tejada et al., 2013) |
| 81 | PSND | Pigment specific normalised difference | $(R_{800} - R_{470})/(R_{800} + R_{470})$ | (Blackburn, 1998) |
| 82 | PSRI | Plant Senescence Reflectance Index | $(R_{678} - R_{500})/R_{750}$ | (Merzlyak et al., 1999) |
| 83 | PSSR | Pigment specific simple ratio | $R_{800}/R_{635}$ | (Blackburn, 1998) |

| | | | | |
|---|---|---|---|---|
| 84 | PWI | Plant Water Index | $R_{900}/R_{970}$ | (Peñuelas et al., 1997) |
| 85 | RDVI | Renormalized Difference Vegetation Index | $(R_{800} - R_{670})/SQRT(R_{800} + R_{670})$ | (Roujean and Breon, 1995) |
| 86 | REP | Red-Edge Position | $700 + 40((R_{670} + R_{780})/2 – R_{700})/(R_{740} – R_{700}))$ | (Guyot et al., 1988) |
| 87 | REP_LE | - | Red-edge position through linear extrapolation | (Cho and Skidmore, 2006) |
| 88 | REP_Li | - | $R_{re} = (R_{670} + R_{780})/2$<br>$700 + 40 * ((R_{re} - R_{700})/(R_{740} - R_{700}))$ | (Guyot et al., 1988) |
| 89 | RGI | Red/Green Index | $R_{690}/R_{550}$ | (Zarco-Tejada et al., 2005) |
| 90 | SAVI | Soil Adjusted Vegetation Index | $(1 + L) * (R_{800} - R_{670})/(R_{800} + R_{670} + L)$ | (Huete, 1988) |
| 91 | SIPI | Structure Intensive Pigment Index | $(R_{800} - R_{445})/(R_{800} - R_{680})$ | (Peñuelas et al., 1995b) |
| 92 | SIPI2 | Structure Intensive Pigment Index 2 | $(R_{800} - R_{440})/(R_{800} - R_{680})$ | (Peñuelas et al., 1995b) |
| 93 | SIPI3 | Structure Intensive Pigment Index 3 | $(R_{800} - R_{445})/(R_{800} - R_{680})$ | (Peñuelas et al., 1995a) |
| 94 | SPVI | Spectral Polygon Vegetation Index | $0.4 * (3.7 * (R_{800}$<br>$- R_{670}) -1.2 * ((R_{530} - R_{670})^2)^{0.5}$ | (Vincini et al., 2006) |
| 95 | SR | Simple Ratio | $R_{800}/R_{680}$ | (Jordan, 1969) |
| 96 | SR1 | Simple Ratio 1 | $R_{750}/R_{700}$ | (Gitelson and Merzlyak, 1997) |
| 97 | SR2 | Simple Ratio 2 | $R_{752}/R_{690}$ | (Gitelson and Merzlyak, 1997) |
| 98 | SR3 | Simple Ratio 3 | $R_{750}/ R_{550}$ | (Gitelson and Merzlyak, 1997) |
| 99 | SR4 | Simple Ratio 4 | $R_{700}/ R_{670}$ | (McMurtrey et al., 1994) |
| 100 | SR5 | Simple Ratio 5 | $R_{675}/R_{700}$ | (Chappelle et al., 1992) |
| 101 | SR6 | Simple Ratio 6 | $R_{750}/R_{710}$ | (Zarco-Tejada and Miller, 1999) |
| 102 | SR7 | Simple Ratio 7 | $R_{440}/_{R690}$ | (Lichtenthaler et al., 1996) |
| 103 | SR705 | Simple Ratio 705 | $R_{750}/ R_{705}$ | (Castro-Esau et al., 2006) |
| 104 | SR8 | Simple Ratio 8 | $R_{515}/R_{550}$ | (Hernández-Clemente et al., 2012) |
| 105 | SRPI | Simple Ratio Pigment Index | $R_{430}/R_{680}$ | (Peñuelas et al., 1995a) |

| | | | | |
|---|---|---|---|---|
| 106 | Sum_Dr1 | - | $\sum_{i=626}^{795} D1i$ | (Elvidge and Chen, 1995) |
| 107 | Sum_Dr2 | - | $\sum_{i=680}^{780} D1i$ | (Filella and Penuelas, 1994) |
| 108 | TCARI | Transformed Chlorophyll Absorbtion Ratio | $3 * ((R_{700} - R_{670}) - 0.2 * (R_{700} - R_{550}) * (R_{700}/R_{670}))$ | (Haboudane et al., 2002) |
| 109 | TCARI2 | Transformed Chlorophyll Absorbtion Ratio 2 | $3 * ((R_{750} - R_{705}) - 0.2 * (R_{750} - R_{550}) * (R_{750}/R_{705}))$ | (Wu et al., 2008) |
| 110 | TCARI2/OSAVI2 | TCARI2/OSAVI2 | TCARI2/OSAVI2 | (Wu et al., 2008) |
| 111 | TCARI/OSAVI | TCARI/OSAVI | TCARI/OSAVI | (Haboudane et al., 2002) |
| 112 | TGI | Triangular greenness index | $-0.5 * (190 * (R_{670} - R_{550}) – 120 * (R_{670} - R_{480}))$ | (Hunt et al., 2013) |
| 113 | TVI | Transformed Vegetation Index | $0.5 * (120 * (R_{750} - R_{550}) – 200 * (R_{670} - R_{550}))$ | (Broge and Leblanc, 2001) |
| 114 | Vog | Vogelmann indices | $R_{740}/R_{720}$ | (Vogelmann et al., 1993) |
| 115 | Vog2 | Vogelmann indices 2 | $(R_{734} - R_{747})/(R_{715} + R_{726})$ | (Vogelmann et al., 1993) |
| 116 | Vog3 | Vogelmann indices 3 | $D_{715}/D_{705}$ | (Vogelmann et al., 1993) |
| 117 | Vog4 | Vogelmann indices 4 | $(R_{734} - R_{747})/(R_{715} + R_{720})$ | (Vogelmann et al., 1993) |
| 118 | VS | Vegetation Stress ratio | $R_{725}/ R_{702}$ | (White et al., 2008) |
| 119 | WI | Water Index | $R_{900}/R_{970}$ | (Peñuelas et al., 1993) |

$R_x$ - reflectance at wavelength x nm.

$D_x$ - derivative of the reflectance spectrum at wavelength x nm.

- No original index name or abbreviation found

## References


Agapiou, A., Hadjimitsis, D., and Alexakis, D. (2012). Evaluation of Broadband and Narrowband Vegetation Indices for the Identification of Archaeological Crop Marks. *Remote Sensing* 4(12)**,** 3892-3919. doi: 10.3390/rs4123892.

Apan, A., Held, A., Phinn, S., and Markley, J. (2004). Detecting sugarcane 'orange rust' disease using EO-1 Hyperion hyperspectral imagery. *International Journal of Remote Sensing* 25(2)**,** 489-498. doi: 10.1080/01431160310001618031.

Ashourloo, D., Mobasheri, M., and Huete, A. (2014). Developing Two Spectral Disease Indices for Detection of Wheat Leaf Rust (Pucciniatriticina). *Remote Sensing* 6(6)**,** 4723-4740. doi: 10.3390/rs6064723.

Blackburn, G.A. (1998). Spectral indices for estimating photosynthetic pigment concentrations: A test using senescent tree leaves. *International Journal of Remote Sensing* 19(4)**,** 657-675. doi: 10.1080/014311698215919.

Boochs, F., Kupfer, G., Dockter, K., and Kühbauch, W. (1990). Shape of the red edge as vitality indicator for plants. *International Journal of Remote Sensing* 11(10)**,** 1741-1753. doi: 10.1080/01431169008955127.

Broge, N.H., and Leblanc, E. (2001). Comparing prediction power and stability of broadband and hyperspectral vegetation indices for estimation of green leaf area index and canopy chlorophyll density. *Remote Sensing of Environment* 76(2)**,** 156-172. doi: 10.1016/s0034-4257(00)00197-8.

Carter, G.A. (1994). Ratios of leaf reflectances in narrow wavebands as indicators of plant stress. *International Journal of Remote Sensing* 15(3)**,** 697-703. doi: 10.1080/01431169408954109.

Castro-Esau, K., Sanchez-Azofeifa, G., and Rivard, B. (2006). Comparison of spectral indices obtained using multiple spectroradiometers. *Remote Sensing of Environment* 103(3)**,** 276-288. doi: 10.1016/j.rse.2005.01.019.

Chappelle, E.W., Kim, M.S., and McMurtrey, J.E. (1992). Ratio analysis of reflectance spectra (RARS): An algorithm for the remote estimation of the concentrations of chlorophyll A, chlorophyll B, and carotenoids in soybean leaves. *Remote Sensing of Environment* 39(3)**,** 239-247. doi: 10.1016/0034-4257(92)90089-3.

Chen, J.M., and Cihlar, J. (1996). Retrieving leaf area index of boreal conifer forests using Landsat TM images. *Remote Sensing of Environment* 55(2)**,** 153-162. doi: 10.1016/0034-4257(95)00195-6.

Cho, M.A., and Skidmore, A.K. (2006). A new technique for extracting the red edge position from hyperspectral data: The linear extrapolation method. *Remote Sensing of Environment* 101(2)**,** 181-193. doi: 10.1016/j.rse.2005.12.011.

Dash, J., and Curran, P.J. (2004). The MERIS terrestrial chlorophyll index. *International Journal of Remote Sensing* 25(23)**,** 5403-5413. doi: 10.1080/0143116042000274015.

Datt, B. (1998). Remote Sensing of Chlorophyll a, Chlorophyll b, Chlorophyll a+b, and Total Carotenoid Content in Eucalyptus Leaves. *Remote Sensing of Environment* 66(2)**,** 111-121. doi: 10.1016/s0034-4257(98)00046-7.

Datt, B. (1999). Visible/near infrared reflectance and chlorophyll content in Eucalyptus leaves. *International Journal of Remote Sensing* 20(14)**,** 2741-2759. doi: 10.1080/014311699211778.

Daughtry, C., Walthall, C., Kim, M., De Colstoun, E.B., and McMurtrey Iii, J. (2000). Estimating corn leaf chlorophyll concentration from leaf and canopy reflectance. *Remote sensing of Environment* 74(2)**,** 229-239. doi: 10.1016/S0034-4257(00)00113-9.

Elvidge, C.D., and Chen, Z. (1995). Comparison of broad-band and narrow-band red and near-infrared vegetation indices. *Remote Sensing of Environment* 54(1)**,** 38-48. doi: 10.1016/0034-4257(95)00132-k.

Filella, I., Amaro, T., Araus, J.L., and Penuelas, J. (1996). Relationship between photosynthetic radiation-use efficiency of barley canopies and the photochemical reflectance index (PRI). *Physiologia Plantarum* 96(2)**,** 211-216. doi: 10.1111/j.1399-3054.1996.tb00204.x.

Filella, I., and Penuelas, J. (1994). The red edge position and shape as indicators of plant chlorophyll content, biomass and hydric status. *International Journal of Remote Sensing* 15(7)**,** 1459-1470. doi: 10.1080/01431169408954177.

Filella, I., Serrano, L., Serra, J., and Peñuelas, J. (1995). Evaluating Wheat Nitrogen Status with Canopy Reflectance Indices and Discriminant Analysis. *Crop Science* 35(5)**,** 1400-1405. doi: 10.2135/cropsci1995.0011183X003500050023x.

Gamon, J.A., Serrano, L., and Surfus, J.S. (1997). The photochemical reflectance index: an optical indicator of photosynthetic radiation use efficiency across species, functional types, and nutrient levels. *Oecologia* 112(4)**,** 492-501. doi: 10.1007/s004420050337.

Gandia, S., Fernández, G., García, J., and Moreno, J. (2004). Retrieval of vegetation biophysical variables from CHRIS/PROBA data in the SPARC campaign. *Esa Sp* 578**,** 40-48.

Garrity, S.R., Eitel, J.U.H., and Vierling, L.A. (2011). Disentangling the relationships between plant pigments and the photochemical reflectance index reveals a new approach for remote estimation of carotenoid content. *Remote Sensing of Environment* 115(2)**,** 628-635. doi: 10.1016/j.rse.2010.10.007.

Gitelson, A., and Merzlyak, M.N. (1994). Quantitative estimation of chlorophyll-a using reflectance spectra: Experiments with autumn chestnut and maple leaves. *Journal of Photochemistry and Photobiology B: Biology* 22(3)**,** 247-252. doi: 10.1016/1011-1344(93)06963-4.

Gitelson, A.A., Buschmann, C., and Lichtenthaler, H.K. (1999). The Chlorophyll Fluorescence Ratio F735/F700 as an Accurate Measure of the Chlorophyll Content in Plants. *Remote Sensing of Environment* 69(3)**,** 296-302. doi: 10.1016/s0034-4257(99)00023-1.

Gitelson, A.A., Gritz, Y., and Merzlyak, M.N. (2003). Relationships between leaf chlorophyll content and spectral reflectance and algorithms for non-destructive chlorophyll assessment in higher plant leaves. *J Plant Physiol* 160(3)**,** 271-282. doi: 10.1078/0176-1617-00887.

Gitelson, A.A., Kaufman, Y.J., and Merzlyak, M.N. (1996). Use of a green channel in remote sensing of global vegetation from EOS-MODIS. *Remote Sensing of Environment* 58(3)**,** 289-298. doi: 10.1016/s0034-4257(96)00072-7.

Gitelson, A.A., and Merzlyak, M.N. (1997). Remote estimation of chlorophyll content in higher plant leaves. *International Journal of Remote Sensing* 18(12)**,** 2691-2697. doi: 10.1080/014311697217558.

Gitelson, A.A., and Merzlyak, M.N. (1998). Remote sensing of chlorophyll concentration in higher plant leaves. *Advances in Space Research* 22(5)**,** 689-692. doi: 10.1016/s0273-1177(97)01133-2.

Gitelson, A.A., Merzlyak, M.N., and Chivkunova, O.B. (2001). Optical properties and nondestructive estimation of anthocyanin content in plant leaves. *Photochem Photobiol* 74(1)**,** 38-45. doi: 10.1562/0031-8655(2001)0740038OPANEO2.0.CO2.

Gitelson, A.A., Zur, Y., Chivkunova, O.B., and Merzlyak, M.N. (2002). Assessing carotenoid content in plant leaves with reflectance spectroscopy. *Photochem Photobiol* 75(3)**,** 272-281. doi: 10.1562/0031-8655(2002)0750272ACCIPL2.0.CO2.

Guyot, G., Baret, F., and Major, D. (1988). High spectral resolution: Determination of spectral shifts between the red and the near infrared. *International Archives of Photogrammetry and Remote Sensing* 11(750-760)**,** 750-760.

Haboudane, D., Miller, J.R., Pattey, E., Zarco-Tejada, P.J., and Strachan, I.B. (2004). Hyperspectral vegetation indices and novel algorithms for predicting green LAI of crop canopies: Modeling and validation in the context of precision agriculture. *Remote sensing of environment* 90(3)**,** 337-352. doi: 10.1016/j.rse.2003.12.013.

Haboudane, D., Miller, J.R., Tremblay, N., Zarco-Tejada, P.J., and Dextraze, L. (2002). Integrated narrow-band vegetation indices for prediction of crop chlorophyll content for application to precision agriculture. *Remote Sensing of Environment* 81(2-3)**,** 416-426. doi: 10.1016/s0034-4257(02)00018-4.

Hernández-Clemente, R., Navarro-Cerrillo, R.M., Suárez, L., Morales, F., and Zarco-Tejada, P.J. (2011). Assessing structural effects on PRI for stress detection in conifer forests. *Remote Sensing of Environment* 115(9)**,** 2360-2375. doi: 10.1016/j.rse.2011.04.036.

Hernández-Clemente, R., Navarro-Cerrillo, R.M., and Zarco-Tejada, P.J. (2012). Carotenoid content estimation in a heterogeneous conifer forest using narrow-band indices and PROSPECT+DART simulations. *Remote Sensing of Environment* 127**,** 298-315. doi: 10.1016/j.rse.2012.09.014.

Huete, A.R. (1988). A soil-adjusted vegetation index (SAVI). *Remote Sensing of Environment* 25(3)**,** 295-309. doi: 10.1016/0034-4257(88)90106-x.

Huete, A.R., Liu, H.Q., Batchily, K., and Van Leeuwen, W. (1997). A comparison of vegetation indices over a global set of TM images for EOS-MODIS. *Remote Sensing of Environment* 59(3)**,** 440-451. doi: 10.1016/s0034-4257(96)00112-5.

Hunt, E.R., Doraiswamy, P.C., McMurtrey, J.E., Daughtry, C.S.T., Perry, E.M., and Akhmedov, B. (2013). A visible band index for remote sensing leaf chlorophyll content at the canopy scale. *International Journal of Applied Earth Observation and Geoinformation* 21**,** 103-112. doi: 10.1016/j.jag.2012.07.020.

Jordan, C.F. (1969). Derivation of Leaf-Area Index from Quality of Light on the Forest Floor. *Ecology* 50(4)**,** 663-666. doi: 10.2307/1936256.

Kim, M.S., Daughtry, C., Chappelle, E., McMurtrey, J., and Walthall, C. (1994). The use of high spectral resolution bands for estimating absorbed photosynthetically active radiation (A par). *CNES, Proceedings of 6th International Symposium on Physical Measurements and Signatures in Remote Sensing***,** 299-306.

le Maire, G., François, C., and Dufrêne, E. (2004). Towards universal broad leaf chlorophyll indices using PROSPECT simulated database and hyperspectral reflectance measurements. *Remote Sensing of Environment* 89(1)**,** 1-28. doi: 10.1016/j.rse.2003.09.004.

le Maire, G., Francois, C., Soudani, K., Berveiller, D., Pontailler, J., Breda, N., et al. (2008). Calibration and validation of hyperspectral indices for the estimation of broadleaved forest leaf chlorophyll content, leaf mass per area, leaf area index and leaf canopy biomass. *Remote Sensing of Environment* 112(10)**,** 3846-3864. doi: 10.1016/j.rse.2008.06.005.

Lehnert, L., Meyer, H., and Bendix, J. (2016). hsdar: Manage, analyse and simulate hyperspectral data in R. *R Package Version 0.4* 1.

Lichtenthaler, H.K., Lang, M., Sowinska, M., Heisel, F., and Miehé, J.A. (1996). Detection of Vegetation Stress Via a New High Resolution Fluorescence Imaging System. *Journal of Plant Physiology* 148(5)**,** 599-612. doi: 10.1016/s0176-1617(96)80081-2.

Maccioni, A., Agati, G., and Mazzinghi, P. (2001). New vegetation indices for remote measurement of chlorophylls based on leaf directional reflectance spectra. *J Photochem Photobiol B* 61(1-2)**,** 52-61. doi: 10.1016/S1011-1344(01)00145-2.

Main, R., Cho, M.A., Mathieu, R., O'Kennedy, M.M., Ramoelo, A., and Koch, S. (2011). An investigation into robust spectral indices for leaf chlorophyll estimation. *ISPRS Journal of Photogrammetry and Remote Sensing* 66(6)**,** 751-761. doi: 10.1016/j.isprsjprs.2011.08.001.

McMurtrey, J.E., Chappelle, E.W., Kim, M.S., Meisinger, J.J., and Corp, L.A. (1994). Distinguishing nitrogen fertilization levels in field corn (Zea mays L.) with actively induced fluorescence and passive reflectance measurements. *Remote Sensing of Environment* 47(1)**,** 36-44. doi: 10.1016/0034-4257(94)90125-2.

Merzlyak, M.N., Gitelson, A.A., Chivkunova, O.B., and Rakitin, V.Y.U. (1999). Non-destructive optical detection of pigment changes during leaf senescence and fruit ripening. *Physiologia Plantarum* 106(1)**,** 135-141. doi: 10.1034/j.1399-3054.1999.106119.x.

Miller, J.R., Hare, E.W., and Wu, J. (1990). Quantitative characterization of the vegetation red edge reflectance 1. An inverted-Gaussian reflectance model. *International Journal of Remote Sensing* 11(10)**,** 1755-1773. doi: 10.1080/01431169008955128.

Oppelt, N., and Mauser, W. (2004). Hyperspectral monitoring of physiological parameters of wheat during a vegetation period using AVIS data. *International Journal of Remote Sensing* 25(1)**,** 145-159. doi: 10.1080/0143116031000115300.

Peñuelas, J., Baret, F., and Filella, I. (1995a). Semi-empirical indices to assess carotenoids/chlorophyll a ratio from leaf spectral reflectance. *Photosynthetica* 31(2)**,** 221-230.

Peñuelas, J., Filella, I., Biel, C., Serrano, L., and Save, R. (1993). The reflectance at the 950–970 nm region as an indicator of plant water status. *International journal of remote sensing* 14(10)**,** 1887-1905. doi: 10.1080/01431169308954010.

Peñuelas, J., Filella, I., Lloret, P., Muñoz, F., and Vilajeliu, M. (1995b). Reflectance assessment of mite effects on apple trees. *International Journal of Remote Sensing* 16(14)**,** 2727-2733. doi: 10.1080/01431169508954588.

Peñuelas, J., Gamon, J.A., Fredeen, A.L., Merino, J., and Field, C.B. (1994). Reflectance indices associated with physiological changes in nitrogen- and water-limited sunflower leaves. *Remote Sensing of Environment* 48(2)**,** 135-146. doi: 10.1016/0034-4257(94)90136-8.

Peñuelas, J., Pinol, J., Ogaya, R., and Filella, I. (1997). Estimation of plant water concentration by the reflectance Water Index WI (R900/R970). *International Journal of Remote Sensing* 18(13)**,** 2869-2875. doi: 10.1080/014311697217396.

Qi, J., Chehbouni, A., Huete, A.R., Kerr, Y.H., and Sorooshian, S. (1994). A modified soil adjusted vegetation index. *Remote Sensing of Environment* 48(2)**,** 119-126. doi: 10.1016/0034-4257(94)90134-1.

Rondeaux, G., Steven, M., and Baret, F. (1996). Optimization of soil-adjusted vegetation indices. *Remote Sensing of Environment* 55(2)**,** 95-107. doi: 10.1016/0034-4257(95)00186-7.

Roujean, J.-L., and Breon, F.-M. (1995). Estimating PAR absorbed by vegetation from bidirectional reflectance measurements. *Remote Sensing of Environment* 51(3)**,** 375-384. doi: 10.1016/0034-4257(94)00114-3.

Sims, D.A., and Gamon, J.A. (2002). Relationships between leaf pigment content and spectral reflectance across a wide range of species, leaf structures and developmental stages. *Remote Sensing of Environment* 81(2-3)**,** 337-354. doi: 10.1016/s0034-4257(02)00010-x.

Smith, R.C.G., Adams, J., Stephens, D.J., and Hick, P.T. (1995). Forecasting wheat yield in a Mediterranean-type environment from the NOAA satellite. *Australian Journal of Agricultural Research* 46(1)**,** 113-125. doi: 10.1071/ar9950113.

Thenkabail, P.S., Smith, R.B., and De Pauw, E. (2000). Hyperspectral Vegetation Indices and Their Relationships with Agricultural Crop Characteristics. *Remote Sensing of Environment* 71(2)**,** 158-182. doi: 10.1016/s0034-4257(99)00067-x.

Tucker, C.J. (1979). Red and photographic infrared linear combinations for monitoring vegetation. *Remote Sensing of Environment* 8(2)**,** 127-150. doi: 10.1016/0034-4257(79)90013-0.

Vincini, M., Frazzi, E., and D'Alessio, P. (Year). "Angular dependence of maize and sugar beet VIs from directional CHRIS/Proba data", in: *Proc. 4th ESA CHRIS PROBA Workshop*), 19-21.

Vogelmann, J.E., Rock, B.N., and Moss, D.M. (1993). Red edge spectral measurements from sugar maple leaves. *International Journal of Remote Sensing* 14(8)**,** 1563-1575. doi: 10.1080/01431169308953986.

White, D., Williams, M., and Barr, S. (2008). Detecting sub-surface soil disturbance using hyperspectral first derivative band rations of associated vegetation stress. *Int. Arch. Photogramm. Remote Sens. Spat. Inf. Sci* 37**,** 243-248.

Wu, C., Niu, Z., Tang, Q., and Huang, W. (2008). Estimating chlorophyll content from hyperspectral vegetation indices: Modeling and validation. *Agricultural and Forest Meteorology* 148(8-9)**,** 1230-1241. doi: 10.1016/j.agrformet.2008.03.005.

Wu, W. (2014). The Generalized Difference Vegetation Index (GDVI) for Dryland Characterization. *Remote Sensing* 6(2)**,** 1211-1233. doi: 10.3390/rs6021211.

Zarco-Tejada, P., Berjon, A., Lopezlozano, R., Miller, J., Martin, P., Cachorro, V., et al. (2005). Assessing vineyard condition with hyperspectral indices: Leaf and canopy reflectance simulation in a row-structured discontinuous canopy. *Remote Sensing of Environment* 99(3)**,** 271-287. doi: 10.1016/j.rse.2005.09.002.

Zarco-Tejada, P.J., González-Dugo, V., Williams, L.E., Suárez, L., Berni, J.A.J., Goldhamer, D., et al. (2013). A PRI-based water stress index combining structural and chlorophyll effects: Assessment using diurnal narrow-band airborne imagery and the CWSI thermal index. *Remote Sensing of Environment* 138**,** 38-50. doi: 10.1016/j.rse.2013.07.024.

Zarco-Tejada, P.J., and Miller, J.R. (1999). Land cover mapping at BOREAS using red edge spectral parameters from CASI imagery. *Journal of Geophysical Research: Atmospheres* 104(D22)**,** 27921-27933. doi: 10.1029/1999jd900161.

Zarco-Tejada, P.J., Pushnik, J.C., Dobrowski, S., and Ustin, S.L. (2003). Steady-state chlorophyll a fluorescence detection from canopy derivative reflectance and double-peak red-edge effects. *Remote Sensing of Environment* 84(2)**,** 283-294. doi: 10.1016/s0034-4257(02)00113-x.
